# Software-assisted dosimetry in peptide receptor radionuclide therapy with ^177^Lutetium-DOTATATE for various imaging scenarios

**DOI:** 10.1371/journal.pone.0187570

**Published:** 2017-11-06

**Authors:** Dennis Kupitz, Christoph Wetz, Heiko Wissel, Florian Wedel, Ivayla Apostolova, Thekla Wallbaum, Jens Ricke, Holger Amthauer, Oliver S. Grosser

**Affiliations:** 1 Department of Radiology and Nuclear Medicine, University Hospital Magdeburg A.ö.R., Otto-von-Guericke University Magdeburg, Magdeburg, Germany; 2 Department of Nuclear Medicine, Charité—Universitätsmedizin Berlin, Berlin, Germany; 3 Department of Nuclear Medicine, University Medical Center Hamburg UKE, Hamburg, Germany; 4 Department of Clinical Radiology, Ludwig-Maximilians-University LMU, München, Germany; University of Nebraska Medical Center, UNITED STATES

## Abstract

In peptide receptor radionuclide therapy (PRRT) of patients with neuroendocrine neoplasias (NENs), intratherapeutic dosimetry is mandatory for organs at risk (e.g. kidneys) and tumours. We evaluated commercial dosimetry software (Dosimetry Toolkit) using varying imaging scenarios, based on planar and/or tomographic data, regarding the differences in calculated organ/tumour doses and the use for clinical routines. A total of 16 consecutive patients with NENs treated by PRRT with ^177^Lu-DOTATATE were retrospectively analysed. Single-photon emission computed tomography (SPECT)/low-dose computed tomography (CT) of the thorax and abdomen and whole body (WB) scintigraphy were acquired up to 7 days p.i. (at a maximum of five imaging time points). Different dosimetric scenarios were evaluated: (1) a multi-SPECT-CT scenario using SPECT/CT only; (2) a planar scenario using WB scintigraphy only; and (3) a hybrid scenario using WB scintigraphy in combination with a single SPECT/low-dose CT. Absorbed doses for the kidneys, liver, spleen, lungs, bladder wall and tumours were calculated and compared for the three different scenarios. The mean absorbed dose for the kidneys estimated by the multi-SPECT-CT, the planar and the hybrid scenario was 0.5 ± 0.2 Sv GBq^-1^, 0.8 ± 0.4 Sv GBq^-1^ and 0.6 ± 0.3 Sv GBq^-1^, respectively. The absorbed dose for the residual organs was estimated higher by the planar scenario compared to the multi-SPECT-CT or hybrid scenario. The mean absorbed tumour doses were 2.6 ± 1.5 Gy GBq^-1^ for the multi-SPECT-CT, 3.1 ± 2.2 Gy GBq^-1^ for the hybrid scenario and 5.3 ± 6.3 Gy GBq^-1^ for the planar scenario. SPECT-based dosimetry methods determined significantly lower kidney doses than the WB scintigraphy-based method. Dosimetry based completely on SPECT data is time-consuming and tedious. Approaches combining SPECT/CT and WB scintigraphy have the potential to ensure compromise between accuracy and user-friendliness.

## Introduction

Peptide receptor radionuclide therapy (PRRT) with radiolabelled somatostatin analogues is an established treatment option for internal radiation therapy in patients with neuroendocrine neoplasias (NENs) [1,2]. Currently, PRRT is performed using ^177^Lu-labelled pharmaceuticals, because of the physical properties of the radionuclide (e.g. emission types and energy, particle range in tissue and physical half-life) [3,4] in treatment of NENs [1,5].

In addition to the beneficial effect on tumours, there are also risks associated with PRRT mostly because of radiation toxicity to tumour-unaffected tissues, especially for kidneys and red bone marrow. To avoid treatment-related side effects, dosimetry is mandatory for PRRT for each treatment cycle. For individualised dosimetry, a variety of factors, for example, tumour size, organ size, uptake and tracer kinetic, should be considered [6–8].

Clinical dosimetry is often performed by evaluating accumulated activity in target regions using a region of interest (ROI)-based evaluation of planar (2D) whole-body (WB) scintigraphy. Next, data from ROI analysis were analysed according to medical internal radiation dose (MIRD) formalism [9,10]. In 2D, the overlapping of regions (e.g. organs) or neglecting additional individual factors (e.g. organ mass [11]) may lead to incorrect determination of the ROI uptake [12], resulting in an over- or underestimation of the absorbed dose in the corresponding region. By using single-photon emission computed tomography (SPECT), the uptake in the ROI is determined without superimposed structures. The combination with a low-dose computed tomography (CT) for morphological mapping and for attenuation correction (CTAC) enables even further improvement in dosimetry, for example, correction for scattered photons [4,12–20].

However, it should be possible to perform individual dosimetry of PRRT patients in a clinical routine using an integrated patient-friendly workflow. For this reason, this study aimed to optimise the dosimetry workflow in PRRT examining three different imaging scenarios for dosimetry: (1) a multi-SPECT-CT scenario using SPECT/low-dose CT only; (2) a planar scenario using WB scintigraphy only; and (3) a hybrid scenario using WB scintigraphy in combination with a single SPECT/low-dose CT. We compared intra-individually the calculated organ and tumour doses obtained by the different imaging protocols and the calculation methodologies implemented by a specific software tool certified for clinical dosimetry. Additionally, we assessed the processing time for the investigator (e.g. physician or physicist) for a single dosimetric evaluation.

## Methods

### Patients

Sixteen consecutive patients with NENs (nine male, seven female, aged 65.6 ± 9.7 yr, 51–82 yr) referred between December 2011 and December 2015 for PRRT with ^177^Lu-[DOTA^0^,Tyr^3^]octreotate (^177^Lu-DOTATATE) were retrospectively analysed. The total number of PRRT cycles was 47 (median = 3, range = 1–4). For the study, we included imaging data of the first therapy cycle of each patient with their specific physique and accumulation pattern (n = 16). PRRT was performed in concordance with established clinical guidelines [8,21].

The retrospective analysis (registration number: 77/14, RAD252) was approved by the local institutional ethics committee (Ethics Committee of the University Hospital Magdeburg). All patients had provided written informed consent on the evaluation of their data.

### Imaging

All examinations were performed using a hybrid SPECT/CT (Discovery NM/CT 670, GE Healthcare, Haifa, Israel). The imaging protocol consisted of planar WB scintigraphy and SPECT/low-dose CT of the thorax and abdomen. The protocol included imaging on 5 days between 4 h and 168 h p.i. (post injection), while each patient was examined after 4 h, 24 h and 72 h.

The SPECT gamma camera was equipped with a medium-energy, general propose collimator (MEGP), and all image acquisitions were performed using a single energy window at 208 keV ± 10%. No energy window for scatter correction was applied. Planar WB scintigraphy (anterior and posterior) was acquired with a matrix of 256 x 1024 and a scan speed of 10 cm min^-1^. For each patient, imaging was performed with an individually chosen constant table high and a fixed detector radius, to realise a minimal distance to the patient contour. For subsequent examinations of the same patient, the initial scan parameters were used. For the estimation of the sensitivity of the gamma camera, a ^177^Lu-filled syringe (V = 50 mL, A = 196 ± 27 MBq) was measured. The syringe was scanned at every patient WB examination and was located below the feet of the patient. For SPECT sensitivity, a separate static image of the syringe was necessary to determine the calibration factor [22]. However, it was also possible to determine the sensitivity factor in concordance with NEMA procedure [23]. Then the corresponding sensitivity factor can be entered manually.

SPECT data were acquired using automatic body contouring with a total 60 angular views (30 per detector) at steps of 6° (30 s/projections) and a 256 × 256 matrix (pixel size = 2.21 x 2.21 mm, zoom = 1.0). SPECT data were acquired for two bed positions covering the thorax and abdomen. Low-dose CT imaging was performed with a primary collimation of 16 x 1.25 mm, pitch = 1.375, rotation time = 0.8 s and an X-ray tube voltage of 120 kVp. The X-ray tube current was 40 mA at the first imaging time point (after 4 h p.i.) and 20 mA for the later four imaging time points. The CT scans with 40 mA and 20 mA were used for the CTAC of the corresponding SPECT data and for the registration of different imaging time points. The CT scans with 40 mA were further used for morphologic-oriented ROI definition. CT data were reconstructed with a matrix of 512 x 512 (pixel size = 0.98 x 0.98 mm) and a slice thickness of 3.75 mm by an iterative CT image reconstruction algorithm (adaptive statistical image reconstruction, ASIR) with an ASIR level of 100% [24].

### Dose calculation

**Fig 1:**
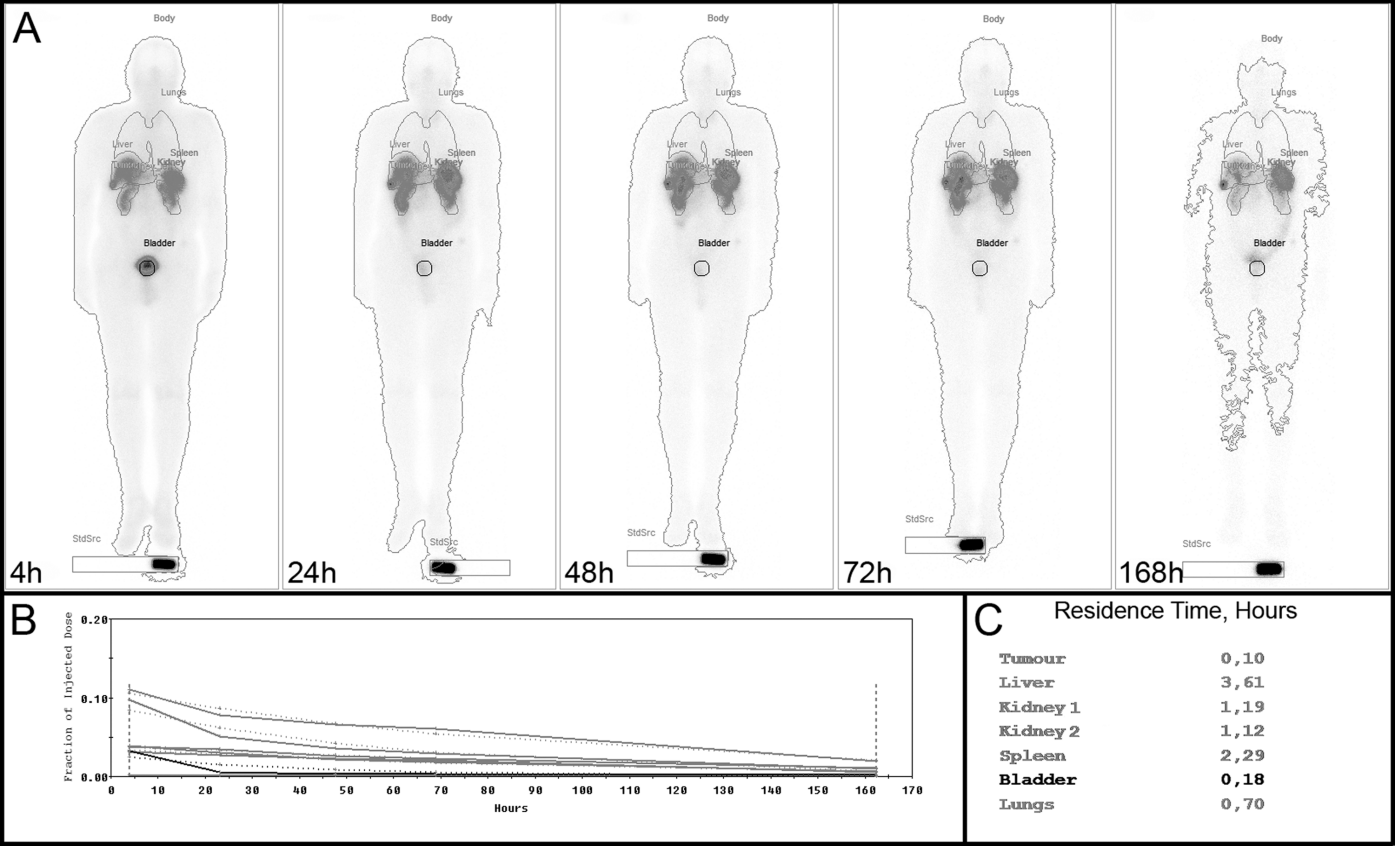
A representative final report of the Dosimetry Toolkit. (A) Illustration of the serial images of a patient with all regions of interest 4 h, 24 h, 48 h, 72 h and 168h p.i. (B) Normalised time-activity curves for all regions of interest. (C) Calculated residence times of all regions of interest.

Analysis was performed with dedicated dosimetry software (Dosimetry Toolkit—DTK, vendor GE Healthcare, Haifa, Israel) for examination of pharmacokinetic uptake and calculation of residence time. Residence time represents the cumulated activity to the organ normalized to the (total) administered activity to the patient according to the MIRD-concept [9]. The DTK provided three different evaluation workflows: (1) based on SPECT/CT imaging (multi-SPECT-CT scenario); (2) based on planar scintigraphy (planar scenario); and (3) based on planar WB scintigraphy with a supplementary single SPECT/CT examination performed in conjunction with one of the planar WB scintigraphies (hybrid scenario). Uptake and residence time were estimated for all patients by each scenario for kidneys, liver, spleen, lungs, bladder content and at least for one liver tumour. Using DTK, a time-activity curve for each previously defined ROI was estimated and fitted automatically by a mono-exponential function for the calculation of residence time. Other analytic fit functions (e.g. bi-exponential function) were not implemented. A final report of the DTK is shown in Fig 1. Using the hybrid scenario, the time-activity curve for each ROI, estimated from sequential planar scintigraphies, was scaled by the activity inside a corresponding volume of interest (VOI) estimated from a single SPECT [22]. Personalized organ dose calculations were performed using OLINDA/EXM software [25] with residence times calculated by DTK.

All dose values calculated by OLINDA/EXM were corrected for gender and adjusted individually for organ/patient weight. Organ masses were calculated from organ volume individually estimated from CT data by using standardized organ densities [26]. Furthermore, the absorbed doses of tumour lesions in the liver were calculated by the density sphere model implemented in OLINDA/EXM [20,27,28] using the volume estimated from CT volumetry and considering a tumour lesion density equal to the liver. All tumour doses were corrected for partial volume effect.

Additionally, CT exposure was analysed by evaluating the dose length product (DLP) documented by the CT. The effective (E) dose for the low-dose CT imaging procedure was calculated by the software CT-Expo [29].

### Multi-SPECT-CT scenario

This scenario is based solely on SPECT/CT data. Image reconstruction was performed by iterative ordered-subset expectation maximisation algorithm (OSEM, 5 iterations, 10 subsets, HANN filter-function with 0.9 cycles/cm) with CTAC. The registration of all scans to one reference scan (the latest SPECT/CT) and the subsequent organ registration were performed by the DTK.

**Fig 2:**
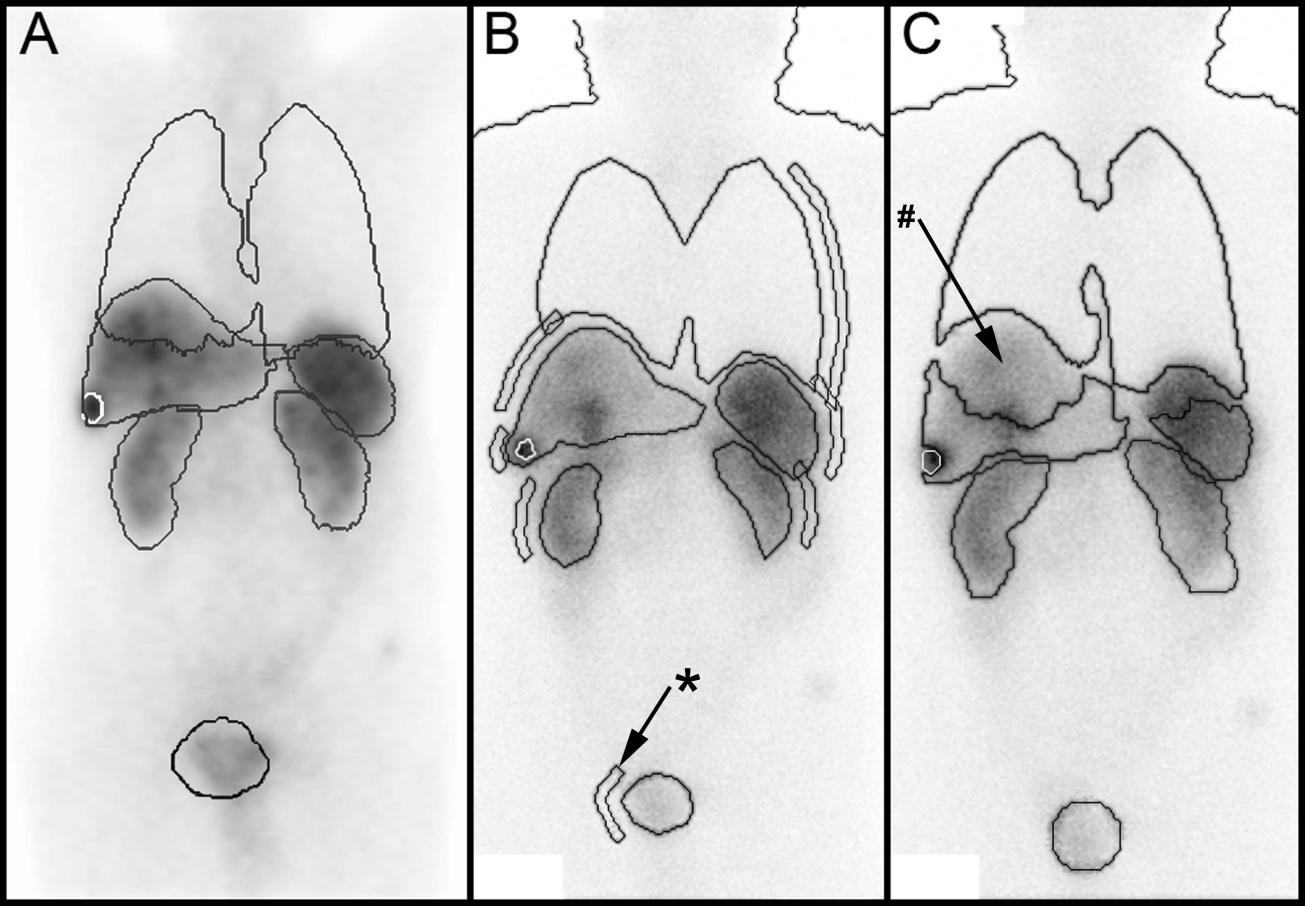
ROI/VOI comparison of all three imaging scenarios 24 h p.i. of the same patient. The delineations of lungs, liver, kidneys, spleen, bladder and tumour (white, in the liver) are shown. (A) 2D presentation (summed coronal slice) of the 3D VOIs of the multi-SPECT-CT scenario. (B) Geometric mean image of the planar scenario with all ROIs. The small elongated delineations (*) next to the ROIs were used for background correction. (C) Geometric mean image of the WB scintigraphies with SPECT/CT based VOIs (hybrid scenario). Here, overlapping regions of interest were automatically removed (#) and corrected.

The segmentation of the VOIs (e.g. for organs and tumours) was performed by automatic, semi-automatic and manual DTK tools using CT or SPECT data. VOI segmentation started with the first SPECT/CT scan (4h p.i.). After defining VOIs for the first SPECT/CT, the VOIs were copied automatically to the residual SPECT/CTs. The VOIs were adjusted for each time point manually if appropriate. Representative VOIs are shown in Fig 2A.

### Planar scenario

Geometric mean images (square root of the product of the counts in the anterior and posterior projections) were automatically calculated from the WB scintigraphies for each time point. The resulting images were co-registered to the first imaging time point (4 h p.i.).

ROIs for body contour and calibration standard (syringe) were automatically defined by the DTK. ROIs for organs and tumours were defined manually with ROI drawing tools for the first imaging time point. After confirming all ROIs, they were automatically copied to the geometric mean images of later time points. Representative ROIs are shown in Fig 2B. Background correction for the count rate was performed for each organ ROI. The corresponding background ROI was defined automatically for each time point next to the organ ROI (Fig 2B). The Background correction for the counts estimated for an organ ROI was applied by subtracting the weighted background counts. Weighting was performed by a factor (wf) respecting the organ thickness [30].

$$wf=1-\frac{d_{organ}}{d_{body}}$$ (1)

The extension of the organ (*d*_organ_) and the body (*d*_body_) in the anterior–posterior direction was previously estimated from CT scans.

### Hybrid scenario

The hybrid scenario combined a series of WB scintigraphies with a single SPECT/CT acquired in a timely manner corresponding to one of the WB scintigraphies. The pre-processing of the scintigraphies is identical to the planar scenario. Furthermore, the SPECT data were reconstructed and a summed coronal slice, representing the reference image, was calculated. The co-registered series of geometric mean WB images were automatically registered to the summed slice.

Furthermore, VOIs (e.g. organs and tumours) were defined by using SPECT/CT data that were acquired nominally 24 h p.i. Finally, the determined VOIs were projected by the DTK to registered geometric mean images. Volumes (or parts of volumes) overlapping in the 2D projection were not considered by DTK for analysis of the geometric mean images (Fig 2C). In these cases, organ activity was corrected for the activity in the removed volume by using the mean activity concentration in the residual volume (VOI) as a surrogate for substitution.

### Inter-observer variability

The influence of observers on ROI definition was examined and inter-observer variability was tested for three independent observers (C.H., H.W. and D.K) defining the kidney ROIs for all patients. All observers were experienced in nuclear medical imaging and clinical dosimetry (4–20 years). The influence of the observer on kidney dosimetry was tested for WB scintigraphy data and for the SPECT/CT data (24 h p.i.). Furthermore, the time required for ROI/VOI delineation was determined.

### Statistics

The R software package (version 3.2.0, R Foundation for Statistical Computing, Vienna, Austria) was used for statistical evaluations. Descriptive parameters were expressed as mean ± standard deviation (SD), median, interquartile range (IQR; 25^th^/75^th^ percentiles) and range. The organ and tumour doses per injected activity calculated by the different scenarios were tested for differences using the Friedman test and Wilcoxon signed-rank test, if applicable. The multi-SPECT-CT scenario represented the standard of truth for this study. Inter-observer variability of the observer in drawing kidneys ROI was examined by Kendall’s coefficient of concordance w_t_. All tests were performed two-sided, and statistical significance was assumed at a *p*-value of < 0.05.

## Results

The mean administered activity of ^177^Lu-Dotatate was A = 7.2 ± 0.4 GBq. The mean weighting factors (wf) used for the planar scenario were: wf_Kidneys_ = 0.82 ± 0.06, wf_Liver_ = 0.52 ± 0.23, wf_Spleen_ = 0.80 ± 0.10, wf_Lung_ = 0.44 ± 0.17, wf_Bladder_ = 0.77 ± 0.12 and wf_Tumour_ = 0.92 ± 0.04.

A detailed summary of the determined organ masses and organ residence times for dosimetry are presented in Table 1 and Table 2. The dose per injected activity (DpA) for each organ is shown in Table 3.

**Table 1:** Patient whole body (WB) weight and determined organ masses. The individual masses estimated with low-dose CT volumetry were used for weight adjustments in OLINDA/EXM software.

	WB (g)	Kidneys (g)	Liver (g)	Spleen (g)	Lungs (g)
**Female**					
mean ± SD	69,000 ± 12,383	400 ± 73	3,338 ± 3,808	461 ± 543	461 ± 128
50^th^ (25^th^/75^th^)	76,000 (57,500/76,500)	407 (354/421)	1,505 (1,442/3,058)	199 (194/395)	619 (553/717)
range	54,000–85,000	307–535	1,092–11,770	170–1,578	458–832
**Male**					
mean ± SD	84,670 ± 13,077	611 ± 104	2,644 ± 797	267 ± 141	829 ± 124
50^th^ (25^th^/75^th^)	90,000 (75,000/94,000)	649 (522/652)	2,777 (2,035/2,968)	233 (213/244)	793 (725/910)
range	66,000–101,000	475–812	1,558–3,922	127–598	681–1,017

Note: Organ mass was calculated by using a standardized tissue density for each organ (kidney = 1.05 g mL^-1^, liver = 1.06 g mL^-1^, spleen = 1.06 g mL^-1^, lungs = 0.26 g mL^-1^) [26].

**Table 2:** Residence times for the first therapy cycle (n = 16 patients) estimated by DTK using different imaging scenarios.

	Kidneys (h)	Liver (h)	Spleen (h)	Lungs (h)	Bladder Content (h)
**Multi-SPECT-CT**					
mean ± SD	2.7 ± 0.8	29.1 ± 43.3	3.3 ± 4.7	0.6 ± 0.5	0.9 ± 0.5
50^th^ (25^th^/75^th^)	2.5 (2.4/3.1)	10.6 (5.4/32.7)	2.4 (1.3/3.1)	0.5 (0.3/0.7)	0.7 (0.5/1.3)
range	1.3–4.3	1.4–165.4	0.7–19.9	0.1–2.3	0.3–1.6
**Hybrid**					
mean ± SD	3.3 ± 1.4	29.4 ± 39.3	3.7 ± 4.3	0.7 ± 0.5	0.7 ± 1.2
50^th^ (25^th^/75^th^)	3.1 (2.40/3.9)	9.0 (6.0/41.2)	2.8 (1.8/3.5)	0.6 (0.3/0.9)	0.3 (0.2/0.4)
range	1.3–6.2	1.1–147.0	0.6–19.3	0.1–2.0	0.1–5.0
*p*^a^	0.0034	n.s.^c^	0.0355	0.5282	n.s.^c^
**Planar**					
mean ± SD	4.1 ± 1.6	23.4 ± 25.0	4.2 ± 2.6	3.0 ± 1.1	1.0 ± 0.8
50^th^ (25^th^/75^th^)	4.3 (3.0/5.3)	9.5 (5.4/46.1)	3.5 (2.9/4.7)	2.9 (2.3/3.7)	0.8 (0.5/1.4)
range	1.3–7.2	2.4–66.8	1.3–12.4	1.0–5.3	0.2–3.1
*p*^b^	0.0017	n.s.^c^	0.0115	< 0.0001	n.s.^c^

^a^Significance of differences between the multi-SPECT-CT and hybrid scenario (Wilcoxon signed-rank test)

^b^Significance of differences between the multi-SPECT-CT and planar scenario (Wilcoxon signed-rank test)

^c^n.s. (not significant) differences between the three groups (Friedman test)

**Table 3:** Dose per injected activity for the first therapy cycle (n = 16 patients), with respect to the different evaluation scenarios.

	Kidneys (Sv GBq^-1^)	Liver (Sv GBq^-1^)	Spleen (Sv GBq^-1^)	Lungs (Sv GBq^-1^)	Bladder Wall (Sv GBq^-1^)
**Multi-SPECT-CT**					
mean ± SD	0.477 ± 0.184	0.663 ± 0.618	0.816 ± 0.406	0.081 ± 0.062	0.199 ± 0.110
50^th^ (25^th^/75^th^)	0.413 (0.325/0.593)	0.375 (0.278/0.935)	0.697 (0.488/1.150)	0.055 (0.039/0.106)	0.173 (0.102/0.301)
range	0.272–0.896	0.080–2.075	0.293–1.584	0.013–0.202	0.061–0.383
**Hybrid**					
mean ± SD	0.588 ± 0.297	0.696 ± 0.624	0.962 ± 0.530	0.130 ± 0.173	0.177 ± 0.335
50^th^ (25^th^/75^th^)	0.503 (0.353/0.819)	0.403 (0.264/1.031)	0.930 (0.516/1.090)	0.086 (0.037/0.139)	0.088 (0.046/0.102)
range	0.295–1.298	0.067–2.332	0.257–2.089	0.017–0.744	0.029–1.362
*p*^a^	0.0052	n.s.^c^	0.0256	0.4332	n.s.^c^
**Planar**					
mean ± SD	0.757 ± 0.433	0.627 ± 0.535	1.429 ± 0.864	0.362 ± 0.141	0.241 ± 0.177
50^th^ (25^th^/75^th^)	0.647 (0.524/0.908)	0.441 (0.288/0.667)	1.136 (0.728/1.906)	0.367 (0.260/0.422)	0.176 (0.136/0.285)
range	0.248–1.823	0.143–1.831	0.576–3.623	0.148–0.698	0.054–0.647
*p*^b^	0.0013	n.s.^c^	0.0020	< 0.0001	n.s.^c^

^a^Significance of differences between the multi-SPECT-CT and hybrid scenario (Wilcoxon signed-rank test)

^b^Significance of differences between the multi-SPECT-CT and planar scenario (Wilcoxon signed-rank test)

^c^n.s. (not significant) differences between the three groups (Friedman test)

There were no significant differences in residence times between the examined three imaging scenarios for the liver (*p* = 0.7788) and the bladder content (*p* = 0.2574). The residence times of the kidneys (*p* = 0.0023), spleen (*p* = 0.0013) and lung (*p* < 0.0001) differed significantly between the three imaging scenarios.

### Dose to organs

The mean absorbed doses for both kidneys were 3.5 ± 1.4 Sv for the multi-SPECT-CT scenario, 4.3 ± 2.2 Sv for the hybrid scenario and 5.5 ± 3.3 Sv for the planar scenario. The organ dose estimated by the scenarios was significantly different (*p* = 0.0023). The median DpA of the kidneys, estimated by the hybrid and planar scenario, was compared to the multi-SPECT-CT scenario and increased by a factor of 1.2 (*p* = 0.0052) and 1.6 (*p* = 0.0013), respectively.

The mean absorbed dose for the liver was 4.8 ± 4.4 Sv for the multi-SPECT-CT scenario, 5.0 ± 4.5 Sv for the hybrid scenario and 4.5 ± 3.8 Sv for the planar scenario. The organ dose calculated by the three different imaging scenarios was not significantly different (*p* = 0.7788).

The calculations for the spleen revealed a mean absorbed dose of 5.9 ± 3.1 Sv for the multi-SPECT-CT scenario, 7.0 ± 4.1 Sv for the hybrid scenario and 10.4 ± 6.5 Sv for the planar scenario. The dose estimated by the three imaging scenarios was significantly different (*p* = 0.0013). The median DpA of the spleen was for the hybrid scenario, which increased by a factor of 1.3 (*p* = 0.0256) and for the planar scenario by a factor of 1.6 (*p* = 0.0020) compared to the multi-SPECT-CT scenario.

For the lungs, mean absorbed doses were 0.6 ± 0.4 Sv for the multi-SPECT-CT scenario, 1.0 ± 1.3 Sv for the hybrid scenario and 2.6 ± 1.1 Sv for the planar scenario. Compared to the multi-SPECT-CT scenario, the median DpA of the hybrid scenario was not significantly different (*p* = 0.4332), but the DpA was significantly higher for the planar scenario with a factor of 6.7 (*p* < 0.0001).

The mean absorbed dose for the bladder wall was 1.5 ± 0.8 Sv for the multi-SPECT-CT scenario, 1.3 ± 2.5 Sv for the hybrid scenario and 1.8 ± 1.4 Sv for the planar scenario. The bladder content volume varied considerably between patients, 318 ± 238 mL (108–954 mL). There were no significant differences between the imaging scenarios, *p* = 0.2574.

### Dose to tumours

Liver tumour lesions (n = 23) were evaluated for each scenario. The tumour masses were 54 ± 87 g (median = 20 g, range = 3–403 g). The details of the tumour doses are shown in Table 4. The mean absorbed tumour doses were 22.1 ± 12.9 Gy for the multi-SPECT-CT scenario, 25.3 ± 17.4 Gy for the hybrid scenario and 45.3 ± 56.8 Gy for the planar scenario. The difference between the scenarios was not significant (*p* = 0.1199).

**Table 4:** Dose per injected activity (DpA) and absorbed tumour doses of n = 23 liver tumour lesions of 16 patients for the first therapy cycle.

	Multi-SPECT-CT	Hybrid	Planar	*p*
**DpA (Gy GBq**^**-1**^**)**				
mean ± SD	2.58 ± 1.47	3.09 ± 2.16	5.32 ± 6.26	
50^th^ (25^th^/75^th^)	2.71 (1.26/3.65)	2.70 (1.34/3.74)	3.13 (2.43/4.65)	0.1199
Range	0.16–5.35	0.20–7.89	0.18–25.30	
**Dose (Gy)**				
mean ± SD	22.08 ± 12.91	25.27 ± 17.37	45.30 ± 56.75	
50^th^ (25^th^/75^th^)	21.70 (11.40/29.75)	24.30 (11.65/29.15)	26.50 (19.75/43.55)	0.1835
Range	1.40–47.60	1.90–59.00	1.60–259.10	

### Inter-observer variability

Definition of 3D kidney VOIs showed a very good inter-observer agreement with respect to the extracted counts, w_t_ = 0.937 (*p* = 0.0003). A good inter-observer agreement was estimated for the definition of 2D kidney ROIs using planar images, w_t_ = 0.634 (*p* = 0.0018). The mean time for drawing kidneys in planar WB scintigraphy was 20 ± 4 s, 15–32 s (C.H.: 24 ± 3 s, D.K.: 18 ± 2 s, and H.W.: 19 ± 2 s). The mean time needed for drawing kidneys in the SPECT/CT data was 151 ± 44 s, 77–316 s (C.H.: 123 ± 33 s, D.K.: 134 ± 13 s, and H.W.: 195 ± 41 s). The time for defining the ROI in WB scintigraphy data was significantly smaller compared to defining the VOI in the SPECT/CT data (*p* < 0.0001).

### CT dose exposure

The effective dose for a single low-dose-CT examination (scan length of 80 cm, thorax and abdomen) was E = 2.6 mSv (male) and 3.2 mSv (female) for a tube current of I = 40 mA (DLP = 172.6 mGy cm) and E = 1.2 mSv (male) and 1.6 mSv (female) for I = 20 mA (DLP = 86.3 mGy cm). A single PRRT cycle yielded an effective dose from CT exposure of E = 7.4 mSv (male) and 9.6 mSv (female) for the multi-SPECT-CT scenario and a CT exposure of E = 2.6 mSv (male) and 3.2 mSv (female) for the hybrid scenario.

## Discussion

In this study, we used two commercially available software tools (DTK and OLINDA/EXM) for semi-automatic evaluation of organ doses in PRRT with ^177^Lu-DOTATATE. We compared dose values for different organs and for tumours estimated by three different scenarios based on SPECT/low-dose CT, planar WB imaging and a hybrid protocol with planar WB imaging and SPECT/low-dose CT imaging. Each scenario differed in terms of accuracy, exposure from additional CT imaging and processing time.

The determined mean DpA of 0.8 ± 0.4 Sv GBq^-1^ for the kidneys per treatment cycle estimated by the planar scenario was comparable to results of other studies using planar WB approaches (range: 0.6–1.2 Gy GBq^-1^) [12,16,19,28,31]. The kidney doses estimated by SPECT/CT-based methods were lower compared to previous results. Garkavij et al. [12] used a hybrid approach in their work, in which planar WB data were scaled by uptake values from SPECT data. They reported an average DpA of 0.8 ± 0.2 Gy GBq^-1^ (our data: 0.6 ± 0.3 Sv GBq^-1^). Authors using multi-SPECT/CT methodology reported a mean kidney dose of 2.6–9.1 Gy per treatment cycle [13,17,18]. Here, our results are comparable to published studies. The DpA calculated for liver by the planar scenario (0.6 ± 0.5 Sv GBq^-1^) exceeds the published values by 0.2 to 0.3 Gy GBq^-1^ [28,31]. In contrast, the mean dose estimated by the multi-SPECT-CT scenario converges with the range of published data of 1.9 to 4.5 Gy [13,17,18]. Both the calculated spleen doses for the planar and the multi-SPECT-CT scenario are in good agreement with the available literature [13,17,18,28,31]. Since the lung and bladder wall in PRRT with ^177^Lu-DOTATATE are not considered as organs at high risk, no corresponding reference values were reported by other authors.

The multi-SPECT-CT scenario provides probably the most accurate data (e.g. for residence times) compared to the planar scenario due to the definition of non-overlapping 3D VOIs by using CT information [32] and the evaluation of CTAC corrected SPECT data [33]. However, the multi-SPECT-CT scenario was highly time-consuming because of the pre-processing of the raw data (reconstruction of SPECT data and co-registration) and the elaborated VOI definition. In addition, the patient accumulated an additional CT exposure from each hybrid SPECT/CT scan. This exposure was significantly smaller for the hybrid scenario using a single hybrid SPECT/CT and was absent for the planar scenario. Using the multi-SPECT-CT scenario for dosimetry, the median DpAs were estimated to be significantly lower for organs (e.g. organs at risk) compared to the planar or hybrid imaging approach. The planar scenario was the fastest and easiest dosimetry scenario due to the simple pre-processing and ROI drawing. The calculated DpAs from the planar scenario were significantly higher for the kidneys, spleen, and lungs and slightly but not significantly increased for the liver and bladder wall compared to the multi-SPECT-CT scenario. The overestimation of the organ doses of the planar 2D imaging scenario compared to the SPECT/CT imaging scenario was due to increased residence times caused by the overlapping segmentation of organ ROIs [32], the non-attenuation corrected data used for dosimetry [33] and the weighting factor for organ thickness for the correction of the count-rate. The lack of consideration of photon attenuation in planar-only imaging methods was probably an important source of error compared to CTAC SPECT-based methods [22]. Especially, in the thoracic region, the lack of attenuation correction led to an overestimation of the lung dose [34]. Furthermore, the high systematic overestimation of the lung dose with the planar scenario by a factor of 6.7 may have been due to the weighting factor of the lungs (wf_Lung_ = 0.44 ± 0.12). As mentioned, this factor was obtained by the ratio of organ thickness to body thickness (Eq 1) and was used to correct the count-rate and therefore the residence time. Since the thickness of the lungs varied widely from the apex to the base and only one weighting factor for thickness per organ can be used in the DTK, it is likely that a reduction of the weighting factor for organ thickness would provide results that are more representative. Compared to the lungs, the kidneys and the spleen have a relatively simple geometric shape, so these weighting factors are probably less error-prone. Unfortunately, an ROI-based background correction to another ROI or a smaller ROI within the target region of the planar data is not provided by the DTK. Such a correction could improve the results of the planar methodology [12]. Furthermore, a correction for the individual organ masses was applied. Using the standard organ masses of OLINDA/EXM, dose calculation can be further distorted resulting in an overestimation of organ dose in our cohort.

In the hybrid scenario, organ VOIs were drawn overlap-free in SPECT or CT data before they were projected onto the planar WB data. A correction for overlapping compartments was performed automatically. Because of the pre-processing and VOI/ROI definition, the time required for the hybrid scenario was less than the time of the multi-SPECT-CT scenario but increased compared to the planar scenario. The calculated DpA did not significantly differ from the multi-SPECT-CT scenario except for the kidneys and the spleen. The difference in results between the multi-SPECT-CT and hybrid scenario could arise from the automatic removal of overlapping ROIs (e.g. right kidney and liver). For the extrapolation of overlapping regions, a uniformly distributed activity in the VOIs was assumed [35]. Inhomogeneous uptake in organs or tumour lesions in the organs might compromise the extrapolation and thus the calculation of the residence time. Another limitation, due to our imaging protocol, occurred when the SPECT data were co-registered to the planar WBs. Here, different patient positioning (e.g. SPECT imaged with arms stretched upwards and WB imaged with arms next to the body) may have led to an internal shift of organs and tumours.

Tumour dosimetry was focused on the liver, since all enrolled patients had NENs with liver-dominant metastases. The dose calculations for the multi-SPECT-CT and hybrid scenario revealed a median DpA of 2.7 Gy GBq^-1^ (multi-SPECT-CT scenario: 22 ± 13 Gy, hybrid scenario: 25 ± 17 Gy). For the planar scenario, a median DpA of 3.1 Gy GBq^-1^ (or 45 ± 57 Gy) was estimated. Other authors reported a comparable tumour dose based on three SPECT/CTs per treatment cycle (21.4 ± 9.7 Gy [17] and 20 Gy [20] with a range of 10–170 Gy [20]). The tumour dose (69 ± 33 Gy) by Gupta et al. [28] determined from planar WB data was larger compared to our planar tumour dose. However, because of heterogeneity in tumours (e.g. somatostatin receptor density and vascularisation) the doses are not necessarily comparable. Furthermore, we used the density sphere model implemented in OLINDA/EXM for tumour dose calculation. This model was based on assumptions (e.g. spherical tumour volume and a homogeneous activity distribution), which can result in erroneous tumour dose calculation. Furthermore, cross-radiation has not been considered [25]. Particularly large tumours probably do not fit the spherical shape and small tumours are compromised by a partial volume effect.

The basic clinical impact of our results is the demonstrated difference in organ doses estimated by various methodologies for organs at risk (e.g. kidneys) in PRRT. Depending on the imaging scenario used for dosimetry, the patients accumulate significantly different kidney doses, which, in turn, affect the number of possible treatment cycles. Patients evaluated with the planar scenario would exceed the tolerance dose of the kidneys (23 Gy [36]) after an average of eight treatment cycles, whilst the accumulated dose calculated by the multi-SPECT-CT scenario is only two-thirds of the reported tolerance. Even the tolerance dose for the kidneys is under discussion according to MIRD 26 [22], so it is either 23 Gy or 27 Gy. Furthermore, higher tolerance doses for the kidneys were proposed (28 Gy and 40 Gy) [22]. Naturally, additional treatment cycles are determined by further parameters (e.g. absorbed dose to the bone marrow). However, with respect to this dose, Sandström et al. [18] demonstrated in their work with 200 patients that the kidneys were the organ at risk in about 98% of all cases. We determined the dose to the red bone marrow by measuring several blood samples with a calibrated NaI-scintillation detector [6]. Because no imaging approach was used for analysis, the data were not published by this study.

Obviously, our dosimetric comparison is restricted to the software provided by the vendor, GE Healthcare, and therefore is subject to the limitations of this particular software package. For example, it is not possible to perform bi-exponential fits for the estimation of the residence time. Even though this would be very useful, especially for the evaluation of specific kinetics (e.g. kidney). This is a limitation that was also reported for actual dosimetric studies (study from Uppsala) [22].

Furthermore, the dead time effects for ^177^Lu-DOTATATE dosimetry are small and a correction was not applied since only the first imaging time point (4 h p.i.) may be effected [22] and dose calculations were performed over 7 days. Nevertheless, the software-assisted workflows simplified and improved essential steps in the internal dosimetry due to automated processing, and the use of specific image editing and segmentation tools (e.g. with correction for overlapping organs). Moreover, a good agreement was observed for organ dosimetry (e.g. kidneys) by using the software toolbox independent of the user’s experience. The CT-based segmentation resulted in an almost perfect agreement of the organ definitions with a similar renal dose determined by the different observers.

The usage of scatter corrections for ^177^Lu imaging is the subject of current discussions. Depending on the collimator, the number of scatter windows, and the width of energy windows, Nijs et al. [37] have shown that the reconstructed scatter corrected counts either under- or overestimated the activity in a lesion (VOI). Our imaging protocol was in accordance with the current MIRD 26 [22] published energy window settings of the study of Uppsala in which no scatter correction was applied.

## Conclusion

SPECT/CT-based dosimetry methods determined significantly lower organ doses compared to the planar scintigraphy method, which was possibly due to the benefit of a CTAC and overlap-free uptake calculation. However, dosimetry based completely on SPECT data is time-consuming and tedious. Hybrid imaging approaches combining SPECT/CT and planar delineation provide a compromise between accuracy and user-friendliness.

## Supporting information

S1 Table Patient demographics and organ weights. (XLS)

S2 Table Residence times and dose values for each organ of each patient. (XLS)

S3 Table Mass, residence times and doses values of the tumours. (XLS)

S4 Table Additional data of the Inter-Observer Variability. (XLS)
